# Autoimmunity in Amyotrophic Lateral Sclerosis: Past and Present

**DOI:** 10.1155/2011/497080

**Published:** 2011-08-01

**Authors:** Mario Rafael Pagani, Laura Elisabeth Gonzalez, Osvaldo Daniel Uchitel

**Affiliations:** ^1^Systems Neuroscience Section, Department of Physiology and Biophysics, School of Medicine, University of Buenos Aires, C1121ABG Buenos Aires, Argentina; ^2^Instituto de Fisiología Biología Molecular y Neurociencias UBA-CONICET and Departamento de Fisiología Biología Molecular y Celular, Facultad de Ciencias Exactas y Naturales, Universidad de Buenos Aires, Ciudad Universitaria, C1428EHA Buenos Aires, Argentina; ^3^Laboratorio de Fisiología y Biología Molecular, Facultad de Ciencias Exactas y Naturales, Universidad de Buenos Aires, Ciudad Universitaria, Pabellón II-2do Piso, C1428EHA Buenos Aires, Argentina

## Abstract

Amyotrophic lateral sclerosis (ALS) is a fatal neurodegenerative disease affecting particularly motor neurons for which no cure or effective treatment is available. Although the cause of ALS remains unknown, accumulative evidence suggests an autoimmune mechanism of pathogenesis. In this paper, we will summarize the current research related to autoimmunity in the sporadic form of ALS and discuss the potential underlying pathogenic mechanisms and perspectives. Presented data supports the view that humoral immune responses against motor nerve terminals can initiate a series of physiological changes leading to alteration of calcium homeostasis. In turn, loss of calcium homeostasis may induce neuronal death through apoptotic signaling pathways. Additional approaches identifying specific molecular features of this hypothesis are required, which will hopefully allow us to develop techniques of early diagnosis and effective therapies.

## 1. Introduction

Amyotrophic lateral sclerosis is a neurodegenerative disorder characterized by a progressive death of motor neurons resulting in fatal paralysis in a few years. ALS was well described by Jean-Martin Charcot in 1869. Since that time, numerous studies have been conducted to characterize the anatomical, physiological, and molecular properties of the disorder [1–4]. A number of genes have been identified in hereditary ALS (named familial ALS), which account for 10% of the cases [5, 6]. The remaining 90% is called sporadic ALS and does not show any conventional hereditary pattern. Similar efforts have been done searching for a therapeutic strategy without success [7–13]. To date, the pathogenic mechanisms of ALS continue being unknown. 

In this paper, we will summarize the current evidence related to autoimmunity in the sporadic form of ALS and discuss the potential underlying pathogenic mechanisms and perspectives.

## 2. Pathogenesis

The mechanisms of the specific neuronal death in ALS are unknown. Nevertheless, numerous observations support the involvement of certain alterations such as an increase in the intracellular Ca^2+^ concentration ([Ca^2+^]_i_) [14–18], excitotoxicity mediated by glutamate [19–22]; generation of free radicals [23–27], and autoimmunity. Recently, more attention has been called to protein inclusions in the cytoplasm of degenerating motoneurons [28]. One of the components of these ubiquitinated aggregates was identified as being TAR DNA-binding protein (TDP)-43 [29, 30], which was found to be mutated in some familial and sporadic ALS patients [31]. 

Although these potentially pathogenic mechanisms are generally investigated separately, it is reasonable to consider that they can be part of a series or parallel events leading to neuronal death. Actually, an increase in [Ca^2+^]_i_ may enhance the generation of free radicals and the release of glutamate and in turn increase [Ca^2+^]_i_ further [32, 33]. Nonetheless, most of the studies of ALS provide evidence of mechanisms associated with the disease but it is not clear whether those alterations are pathogenic or a nonpathogenic epiphenomenon. 

Morphological, biochemical, pharmacological, and physiological studies performed either in animal models, cell culture, or with *ex vivo* preparations support the existence of autoimmune mechanisms in ALS [14–18, 34–38]. Typical hallmarks of autoimmunity such as circulating immune complexes, higher frequency of a particular histocompatibility type, or association with other autoimmune diseases have been reported [39–41].

## 3. Humoral Factors and Antibodies from ALS Patients That Affect Motoneurons

### 3.1. Effect of Sera and Purified Antibodies Using In Vitro and In Vivo Systems

Most studies have been carried out examining the effect of sera or purified antibodies from ALS patients searching for general autoimmune markers aimed at identifying the pathogenic mechanisms, a necessary step towards therapy development. The earliest studies reported that sera from ALS patients induced demyelination, killed or damaged spinal or cerebellar cultured neurons [42–44] whereas Horwich and colleagues [45] did not observe such effects on motoneuron cultures. The interpretation of these data may be difficult because serum is complex and undefined, and the experimental conditions may induce opposite effects regardless of the humoral factors potentially associated with ALS. It is known that cultured cells may be particularly vulnerable to noxious stimuli and that serum applied on cell cultures promotes cellular survival [46]. Other studies also showed that antibodies from ALS patients (ALS-Abs) presented immunoreactivity against myelin [47]. An approach aimed at examining specifically the sera effect and attempting to avoid any unspecific effect owing to the vulnerability of cultured cells was performed by Liveson and colleagues [48]. This study examined the effect of sera on organotypic cultures of spinal cord, and a slight myelinotoxic activity was detected only in 2 of 11 sera tested [48]. An additional study using purified ALS-Abs in organotypic spinal cord cultures showed no changes in the number and morphology of ventral horn neurons after a treatment as long as three weeks with ALS-Abs [49]. Strikingly, these studies examined only the effect of the sera or ALS-Abs on the cell body of motoneurons but not at the motor nerve terminal. Indeed, several alterations in ALS patients have been reported at the synaptic level [50–54] which is consistent with the physiological and morphological alterations reported in the neuromuscular junction (NMJ) from *in vivo* and *ex vivo* mouse and rat preparations [18, 34, 35, 38, 55–58]. 

Muscular fatigue, muscle denervation, and alterations in synaptic transmission, such as a decrease in the frequency and amplitude of the miniature end-plate potentials (MEPPs) and higher quantal content (QC) compared with control individuals, have been consistently observed in patients with ALS [50–52, 59, 60]. Acute treatment of muscles with ALS-Abs increased the frequency of spontaneous and asynchronous transmitter releases in mouse or rat neuromuscular preparations [18, 34, 35, 55, 56, 58]. Although the acute exposure of the neuromuscular preparations to ALS-Abs induced an increase in MEPPs frequency, it is feasible that a longer exposure may result in a reduction of MEPPs frequency as observed in ALS patients [52]. This idea is consistent with studies on the long-term effect of ALS-Abs [35]. Application of ALS-Abs on the *levator auris *muscle by subcutaneous injection during 4 to 12 weeks also increased the frequency of MEPPs in five out of seven ALS-Abs tested, and all of them produced significant changes in the quantal content of evoked neurotransmitter release. In muscles treated with the antibodies from one of the ALS patients, synaptic activity was completely absent and it was consistent with histological studies demonstrating complete denervation. Axonal degeneration and denervation were present in most muscles treated with ALS antibodies, but not in muscles treated with control antibodies [35]. Thus, ALS-Abs appear to lead to long-lasting effects at the neuromuscular junction ranging from an increase of the frequency of MEPPs to complete disruption of neurotransmission. 

Additional electrophysiological studies on mouse muscles treated with ALS-Abs have revealed synaptic changes in the molecular machinery involved in neurotransmission [38]. Neuromuscular transmission, in physiological conditions, is mediated by P/Q-type voltage-dependent calcium channels (VDCC) [61, 62]. However, after several weeks of treatment with ALS-Abs, the neuromuscular transmission is mediated by P/Q-type and L-type VDCC. The L-type VDCC is expressed in motor nerve terminals [63]; however, this channel only participates in synaptic transmission in stages of high synaptic remodeling, such as after treatment with botulinum toxin type A, regenerating NMJ after crash of motor nerve and rodent neonatal muscles where maturation of NMJ takes place [62, 64–66]. The functional coupling of L-type VDCC [38] not only shows that the ALS-Abs may induce functional alterations, but also provides evidence of specific molecular and physiological synaptic modifications associated with NMJ regeneration. This is consistent with denervation and reinnervation changes observed in the SOD1 (superoxide dismutase-1) animal model and ALS patients [54].

The interaction of ALS-Abs with motor nerve terminals was suggested by experiments showing the capability of terminals to preferentially uptake those antibodies, compared with control antibodies in mice [37, 57], and also by the detection of internalized antibodies in spinal cord and motor cortex of patients with ALS and in animal models [67–69]. The internalization of ALS-Abs was expected to occur by interaction with nerve terminals, since uptake by nerve terminals is a normal process for molecules that bind to them, such as tetanus and cholera toxins, antibodies raised against synaptosomes, or inorganic mercury [70–73]. Recently, by using ALS-Abs as primary antibodies in immunofluorescence experiments, it was shown that the IgGs purified from ~50% of the ALS patients presented immunoreactivity against the presynaptic membrane of motor nerve terminals and induced synaptic potentiation [18]. 

Demestre and colleagues have reported that motor neurons are susceptible to apoptosis through activation of caspase 3 when motor neuron cultures are treated with ALS-Abs, compared with control-IgGs [74]. These results are in accordance with previous studies in cell culture systems like the VSC4.1 hybrid motor neuron cell line [75], a human neuroblastoma cell line [76], and primary rat brain and spinal cord cultures [76]; in addition, ALS-Abs enhanced cell death in the A127 glioblastoma cell line [77].

### 3.2. Antibodies against Voltage-Dependent Calcium Channels

Biochemical and electrophysiological evidence suggested that antibodies from 40 to 60% of ALS patients show immunoreactivity against VDCC. For instance, immunoreactivity against L-type VDCC purified from muscle fibers (Ca_v_1.1) and a reduction in Ca^2+^ currents were reported [78–80]. However, these observations may not be underlying the alterations reported on synaptic transmission because of a number of reasons. First, increased neurotransmitter release is expected to be associated with an increase instead of a reduction in Ca^2+^ currents [81, 82]. Second, the cytotoxic effect of the ALS-Abs on cultured motoneurons was inhibited by P/Q- and N-type VDCC blockers, but not L-type blockers [44]. Third, therapeutics trails using L-type VDCC blockers like verapamil or nimodipine did not affect the progression of the disease [83, 84]. Fourth, L-type VDCC is not normally coupled to synaptic transmission [62, 85–87]. However, recent investigations in our laboratory showed that L-type VDCC regulates synaptic vesicle recycling [88], suggesting that even when the channel is not directly coupled to synaptic transmission, it can affect synaptic vesicle availability, and in turn, neurotransmitter release. 

Biochemical evidence from Kimura and colleagues showed that ALS-Abs bind to L-type VDCC and blocks the interaction of the monoclonal antibody (8B7 mAb) against L-type channels, suggesting overlapping epitopes of the two antibodies. However, Nyormoi provided evidence indicating that Kimura's observations were not due to ALS-Abs; he claimed that destruction of the antigen by serine proteases copurified with the IgG prevented the interaction of the 8B7 mAb with the VDCC [89]. Nevertheless, it was shown recently that serine proteases from ALS sera have similar cytopathological effects to those obtained from healthy subjects, which are distinct from those observed after ALS IgG passive transfer [90].

Besides the effect of ALS-Abs linked to L-type channels [78–80], other authors found that ALS-Abs increased P/Q-type Ca^2+^ currents in Purkinje cells [91] or decreased Ca^2+^ currents of cultured granule cells [92] or shifted to the left the activation curve of Ca^2+^ current in cultured rat motoneurons [93]. Thus, these results suggest that ALS-Abs modify Ca^2+^ currents, but the particular effect depends on the cellular type and not on the types of VDCC [93–95]. Hence, it is possible to conclude that somehow ALS-Abs may modify Ca^2+^ currents but it is not clear if the antibodies interact directly with VDCC or modify Ca^2+^currents by interacting with another molecule capable of regulating Ca^2+^ currents. For instance, G-protein-coupled receptors are strong modulators of the family 2 of VDCC (i.e., Ca_v_2.1, Ca_v_2.2, and Ca_v_2.3) [96] and recent evidence shows that receptor tyrosine kinase regulates VDCC through p21-ras and ERK 1/2 [97, 98]. Consistently, biochemical and physiological experiments did not detect immunoreactive ALS-Abs against VDCC [99, 100] or effects on synaptic transmission mediated by interaction with VDCC [18]. 

In support of the existence of antibodies directed against calcium channels or related proteins, our recent studies showed that the absence of P/Q-type channel *α*_1_ subunits reduces ALS-IgG binding to mouse NMJs and suppresses antibody effects on spontaneous acetylcholine release. These experiments further indicate that IgGs from ALS patients would react with a putative protein that depends on the P/Q- type channel presence at the mouse neuromuscular junction (Gonzalez L. et al., unpublished data).

### 3.3. Antibodies against Other Molecular Targets

Different investigations have provided evidence indicating that a proportion of patients with ALS have antibodies directed against other targets such as neurofilaments, Fas receptor (CD95), fetal muscular proteins, and vascular antigens [76, 79, 101–106]. The potential role of those antibodies has not been investigated as much as antibodies potentially directed against VDCC. Actually, the evidence suggests that ALS-Abs may be directed to an unknown molecule that in turn may modify Ca^2+^ currents and Ca^2+^ homeostasis. Thus, since in the SOD1 mutant mice a pathway downstream of Fas is involved in motor neurons death and also these mice show higher sensibility to activate the pathway [107, 108], the Fas receptor could be a candidate to investigate further. Nonetheless, a recent study in this animal model suggested that this familiar form might not be associated with a deregulation in the immune system, as SOD1 mutant mice, which were also B cell deficient (i.e., *μ*MT/SOD1), did not show altered mortality or rotarod performance compared with the single mutant SOD1 [109]. On the other hand, antibodies against neurofilaments, fetal muscular proteins and vascular antigens seem to be more likely markers of an autoimmune disorder owing to the fact that they are directed to intracellular or systemic proteins [110]. Taken together, is clear that a complete screening to identify one or more molecular targets for antibodies from ALS patients is required. The recent development of the proteomics technology will help enormously in this task. The identification of antigens for ALS-Abs is currently underway in our laboratory. The identification of one or more antigenic targets will provide a system to develop a specific ALS animal model and determine the precise role of autoimmunity in ALS as well as therapeutic approaches.

### 3.4. Alteration of Calcium Homeostasis by ALS Antibodies

Besides controversies related to the molecular target of ALS-Abs, calcium homeostasis alterations and associated structural changes were consistently reported. Using immunohistochemistry and electron microscopy techniques, it has been shown that ALS-Abs enhance [Ca^2+^]_i_ and structural alterations in motor nerve terminals as well as in cell bodies of spinal motoneurons after intraperitoneal or intramuscular injection in mice [14, 16, 17]. Of note, ALS-Abs produced dose-dependent calcium increases either in axon terminal synaptic vesicles and mitochondria or in rough endoplasmic reticulum, mitochondria and Golgi. Moreover, increased mitochondrial volume and increased number of synaptic vesicles compared to any of the disease control groups were detected [14–16]. Worth mentioning, similar structural modifications and increased [Ca^2+^]_i_ were observed in motor nerve terminals in muscle biopsies from ALS patients [15]. About 70% of ALS-derived IgGs induced the appearance of a population of motoneurons with electron lucent cytoplasm, distended Golgi, disrupted Nissl's bodies and mitochondria (i.e., necrosis). However, 30% of ALS-IgGs additionally induced electron-dense degeneration in 40% of motoneurons [17]. These neurons exhibited shrinkage and condensed nuclear chromatin and resembled preliminary stages of apoptosis. Owing to the well-known effects of [Ca^2+^]_i_ homeostasis deregulation [111] and the correlation between [Ca^2+^]_i_ increment and structural changes [14, 16, 17], these data suggest that the increased [Ca^2+^]_i_ is responsible for the structural changes. Moreover, the mechanisms underlying increased [Ca^2+^]_i_ may be those detected to enhance synaptic transmission in motor nerve terminals [17]. The activation of this increased neurotransmitter release required a non-constitutive Ca^2+^ influx through N-type (Ca_v_2.2) calcium channels and phospholipase C activity. Once the ALS-Abs-induced synaptic potentiation is started, the calcium influx through VDCC is no longer required. Instead, the activation of the inositol 1,4,5-trisphosphate receptors (IP_3_R) and ryanodine receptors is necessary to both activate and sustain the increased neurotransmitter release [18].

In contrast, no changes in [Ca^2+^]_i_ by ALS-Abs application was observed in synaptosomes from cerebral cortex of rat, measured by Fura 2 Ca^2+^ imaging [112]. These experiments support the hypothesis that ALS-Abs have specific targets and tissue specificity, providing a clear mechanism for the selective vulnerability of motoneurons in ALS. In other words, ALS-Abs may affect [Ca^2+^]_i_ in motor nerve terminals but not in nerve terminals from the cerebral cortex. Other experiments provide additional evidence arguing in favor of tissue specificity. Although ALS-Abs affect motor nerve terminals, both morphologically and physiologically, they do not affect spinal cord organotypic cultures. Similar effects are observed after either intraperitoneal or intramuscular injection of ALS-Abs, and immunoreactivity of ALS-Abs was only reported against NMJ [16–18, 48, 99].

## 4. Animal Models

Autoimmune animal models of ALS have been developed by inoculation of purified motoneurons [67] or spinal cord ventral horn homogenate to Guinea pigs [68, 113]. In both models, inoculated material was obtained from bovine. The experimental autoimmune motor neuron disease (EAMND) results in lower motoneuron signs [67], while the experimental autoimmune gray matter disease (EAGMD) results in both upper and lower motoneuron destruction [68, 113]. To generate the ALS autoimmune models, antigens were injected into Guinea pigs 4 times at 4-week intervals. An increase of the frequency of MEPPs and loss of motoneurons within the spinal cord were observed two months after the last immunization. In this animal model, immunoreactivity against IgG at spinal cord motoneurons and at the endplates were detected, showing accumulation of antibodies and suggesting autoimmunity as a pathogenic mechanism. The animal model originally developed by Engelhardt and colleagues [69] has been recently reproduced in a study that showed convincing evidence of upper and lower motoneuron damage, from which the involvement of oxidative stress in the neuronal degeneration was suggested [113]. The EAGMD is the model that better resembles ALS because it is characterized with loss of motoneurons, the presence of denervation, inflammatory foci within the spinal cord, IgG within motoneurons, and electrophysiological abnormalities of the neuromuscular junction [52, 55, 68, 114, 115]. 

If the mechanisms involved in the ALS animal model and ALS patients are equivalent, the antibodies purified from the ALS animal model should mimic the effect of the antibodies purified from patients. Noticeably, both the antibodies from ALS patients and the ALS animal model injected in mice produced limb weakness, increase of the frequency of MEPPs and presence of immunoreactivity for IgG at the NMJ and motoneuron cell bodies [55]. The similarities between the effects induced by antibodies from the ALS animal model and patients strongly support an autoimmune etiology for this disorder.

## 5. Hallmarks of Autoimmunity in ALS

Since autoimmune mechanisms have been considered as a pathogenic factor in ALS, several laboratories started to look for typical signs or hallmarks of autoimmune diseases such as immunological alterations and associated disorders. The proportion of lymphocyte subsets has been reported to be normal in ALS patients [116]. However, 19% of ALS patients and around 20% of their relatives presented thyroid disease [41]. Furthermore, interesting but controversial observations had been reported. High frequency of the HLA (human leukocyte antigen) A3 and HLA B12 histocompatibility antigens was found in ALS patients with rapid and slow progression, respectively [39], but it was not detected in other patients [117]. Similarly, an increased incidence of immune complexes in serum and kidney was detected by one laboratory [40] but not by another [117].

In ALS, a systemic inflammatory response is not reported. However, there is evidence suggesting that neuroinflammation may be a pathological characteristic of this disease [114, 118–120]. An important marker of autoimmunity is the prevalence and extent of T lymphocytic infiltration in the ventral horn of the spinal cord from ALS patients [114]. Using monoclonal antibodies against macrophages T and B cells, the authors revealed that as high as 79% of the specimens showed a cellular mononuclear infiltration. Tissue samples from other ALS subjects with the same duration and clinical signs did not show lymphocytic infiltration, suggesting that it is unlikely that such infiltrates appear as a consequence of spinal cord atrophy. The cellular composition of the spinal cord inflammatory infiltrate comprises a suppressor/cytotoxic T-cell subset and macrophages (*in the anterior and lateral corticospinal tracts and anterior horns*) [118]. Similarly, Engelhardt and colleagues [119] observed T-helper cells (*in proximity to the corticospinal tract degeneration*) and T-helper and T-suppressor/cytotoxic cells (*in ventral horns*). Other laboratories also observed that inflammation in ALS spinal cord as well as in cortex is based on macrophages and T cells [120]. Additional evidence pointing towards an involvement of autoimmune processes has been the recent finding of increased levels of interleukins IL-17 and IL-23 in serum and cerebrospinal fluid of ALS patients. This increment is thought to be a sign of Th-17 activation, a subset of T cells suggested to be crucial in destructive autoimmunity [121]. 

Recent studies have shown that microglia/macrophage activation and immune reactivity is relatively common in spinal cord tissues of patients with ALS and the SOD1 animal model [122, 123]. The presence of immature and activated-mature dendritic cells (i.e., CD1a and CD83 positive cells) was detected in ventral horn and corticospinal tracts from ALS individuals. Monocytic, macrophage, and microglial transcripts (CD14, CD18, SR-A, and CD68) were increased in ALS spinal cord, and activated CD68(+) cells were shown in close proximity to motor neurons. The expression of the chemokine MCP-1 (monocyte chemotactic protein-1), which attracts monocytes and myeloid dendritic cells, and of the cytokine macrophage-colony stimulating factor (M-CSF) was also increased in ALS tissues. The rate of disease progression positively correlates with the detection of dendritic cell transcripts [122]. This observation along with extensive spinal cord microglial/macrophage activation before the onset of clinical symptoms [124] and prior to evidence of significant motor neuron loss in an ALS mouse model suggests that systemic immune deregulation plays a key role in ALS. 

It is interesting to note the existence of other disorders similar to ALS, such as the ALS-like autoimmune syndromes. The best characterized is the multifocal motor neuropathy (MFMN) [125] that manifests as asymmetric muscular weakness with slow progression affecting preferentially distal muscles. In patients with MFMN, signs of upper motoneuronal compromise are not observed. The diagnosis is confirmed on the base of motor nerve conduction block or an increase in the level of antibodies against ganglioside GM1. These antibodies cause the classical physiological changes in this syndrome [126, 127]. This conclusion is based on the excellent response of the patients to immunosuppressive therapy with cyclophosphamide or intravascular immunoglobulin [125].

## 6. Immunosuppressive Therapy in ALS

The ultimate and paramount objective of the investigation in ALS is to develop a therapeutic treatment. Many different therapeutic trials have been performed; most were based on various hypotheses of mechanisms for neuronal death, including oxidative damage, loss of trophic factor support, glutamate-mediated excitotoxicity, and chronic inflammation [13]. The discovery of a group (~3%) of familial ALS cases involving mutations in the SOD1 gene led to the development of transgenic mouse models widely used for testing possible drugs. The negative results of a variety of approaches highlight the differences between human subjects with ALS and any animal model [128]. Another recent advance with regard to ALS therapeutics was the reported improvement of SOD1 mutant mice as well as sporadic patients under lithium treatment [129]. However, these studies have raised some doubts concerning the design of the clinical trial [130, 131]. Additionally, the results showed in the animal model could not be replicated by another group [132]. So far, no breakthrough has yet occurred in ALS therapeutics and present thinking is that a combination of drugs may be required to effectively slow the multifactorial neurodegenerative process [13].

A therapeutic approach based on the hypotheses of an autoimmune pathogenic process should stop or at least modify disease progression. However, immunosuppressive drugs such as corticosteroids, azathioprine, cyclophosphamide, or combinations of them, as well as plasmapheresis therapy or intravenous immunoglobulins (IVIG), have failed to affect the progression of the disease [7–11]. Sequential treatment with plasmapheresis followed by an immunosuppressant [cyclophosphamide, cyclosporine, and IVIG (Synchronized immunosuppression therapy)] was reported to transiently reduce electrophysiologic abnormalities at the neuromuscular junction of some ALS patients [133]. 

Although these negative results discourage this kind of research, they should be analyzed considering that when the patients are diagnosed with ALS, there are already pathological and physiological evidence that the disease is well established, with massive loss of motor neurons [128]. Furthermore, little is known about the duration of any phase of motor neuronal “sickness,” from which motor neurons could be rescued by an effective therapy. In addition, it must be remembered that the success of any immunosuppressive therapies is strongly affected by the particular kind of autoimmunity, the side effects, and the general condition of patients under treatment [134].

## 7. Autoimmunity as Pathogenic Mechanism in ALS

A large body of evidence suggests the involvement of autoimmunity as a pathogenic mechanism; however, the present evidence data is not conclusive. Taken together, several general conclusions can be drawn. First, the population of patients with sporadic ALS is not homogenous and it is clear that, if autoimmune mechanisms are pathogenic, this finding cannot be generalized to the entire population of sporadic ALS individuals. Second, abundance of research has shown autoimmune mechanisms in ALS; however, it is not clear if the autoimmune alterations are involved in the pathogenesis or just appear as an epiphenomenon, particularly because immunosuppressive therapies have failed to suppress or delay the disease. It is feasible that the antibodies directed against intracellular antigens would not be pathogenic, since antibodies for intracellular proteins appear to be a byproduct of autoimmunity [110]. Experiments with the autoimmune animal models strongly support autoimmunity as a pathogenic mechanism, but an effective therapy is still required as a final proof. Third, the observation that the IgG purified from a group of sporadic ALS patients, but not familial ALS patients, specifically interacts with the presynaptic membrane of motoneurons and modulates synaptic transmission through specific mechanisms also suggests that ALS-Abs can be pathogenic [18]. Fourth, a major concern is that the therapeutic approaches have failed [7–12]. However, it is likely that these immunosuppressive therapies were not appropriate. 

Although it is difficult to establish an effective therapy for any autoimmune disease, such as diabetes [134], in ALS, a few issues such as the age of patients, alimentary difficulties, and the advanced stage of the disease could make more difficult to obtain a positive response to therapy. However, if ALS is caused by autoimmunity, we would expect that drastic immunosuppression by total lymphoid irradiation (TLI) produces an effect in ALS patients [12]. In this study, no change in the progression of the disease was observed. However, the authors found that, as expected, some parameters of immune function were reduced by TLI treatment showing adequacy of the immunosuppression but levels of antibodies against gangliosides GM1 and GDlA were not reduced. These findings illustrate that even the strongest approaches for immunosuppression may have selective effects and do not necessarily inhibit all immune responses [12]. In addition, we and others have documented that immunological mechanisms are capable of triggering long-lasting effects [16–18, 35], suggesting that even in the absence of an active immunological noxious agent (e.g., ALS-Abs), the damage and/or mechanisms leading to physiological deficits are already established, without requirement of additional induction or presence of such unknown pathogenic mechanisms. 

Consistent with this notion, therapeutic trials aimed at reducing oxidative stress and/or excitotoxicity mediated by glutamate did not stop ALS progression [13, 135]. One potential explanation is that, in all cases, when therapeutic trials were performed, the disease was too advanced and may be unsusceptible to be stopped or reversed. For instance, a cyclooxygenase-2 (COX-2) inhibitor prolonged survival in a SOD1 ALS model by slowing disease onset [121] but did not alter progression after onset. Consistently, in a human trial, this COX-2 inhibitor did not alter the progression of ALS [136]. In fact, since it is the motor neuron loss that provides the symptoms for ALS diagnosis [137], when ALS patients are diagnosed, they already have a reduced number of motor neurons [138–141]. This situation implies that investigations on earlier detection will be essential for therapeutic treatment of ALS. 

In accordance with the idea that it is essential to identify early events in the pathogenesis of ALS, next we depict an autoimmune hypothesis focused on potential early events. Briefly, autoantibodies against one or more antigens at motor nerve terminals bind to them triggering a signaling system which includes Ca^2+^ influx and activation of Ryanodine and IP_3_ receptors increasing [Ca^2+^]_i_, which modulates synaptic transmission [18, 34]. Although ALS-Abs may also act in a different subcellular localization of motor neurons, we propose motor nerve terminals as primary and/or earlier target since they are located outside the hematoencephalic barrier and constitute a target for autoimmune response [142]. The constitutive [Ca^2+^]_i_ homeostasis deregulation may lead to endoplasmic reticulum (ER) stress and/or mitochondrial dysfunction with the consequent activation of apoptotic pathways such as caspase 3 [14–17, 74]. These events may lead to denervation and synaptic changes [35, 38, 54]. Additionally, injured neurons through secretion of proinflammatory effectors may activate astrocytes which in turn may promote further mitochondrial damage and apoptosis in motoneurons [143]. Moreover, the hypothesis that the neuronal damage is initiated in nerve terminals is consistent with the observation that ALS is a motor neuron pathology affecting first distal axons in the SOD1 ALS mouse model as well as in ALS patients [54, 143].

## 8. Perspectives

It is clear that there is a need for an effective therapy to interrupt the course of the disorder but also there is a necessity for earlier diagnosis. An effective therapy will alleviate ALS patients and provide the strongest evidence of the mechanisms involved, but an early diagnosis is still required to treat ALS as soon as possible to assure a complete recovery of motor functions. 

Early diagnosis through biological markers seems to be a tendency in ALS research and also a promising one. These studies have discovered specific protein alterations detectable in cerebrospinal fluid and blood as well as in tissues of ALS subjects. High-throughput technologies have revealed sets of proteomic or metabolic biomarkers that can discriminate between ALS and control groups [144, 145]. However, validation of identified candidates is required. 

Matrix metalloproteinases are a family of enzymes essential for organizing the extracellular matrix [146]. The detection of active matrix metalloproteinase-9 (MMP-9) by ELISA (enzyme-linked immunosorbent assay) in serum from patients with ALS and Guillain-Barre syndrome and its correlation with nerve damage suggests that MMP-9 can be used as an early marker in both disorders [147, 148]. 

Gene discovery and system biology tools applied to ALS research seems to be another developing area. Since it is suspected that the sporadic form of ALS is caused by multiple genetic variants that individually make relatively weak contributions to risk, genome-wide screens to asses this hypothesis were proposed. Owing to the fact that this approach requires a large sample size, collaborative efforts have created a public repository of human DNA, immortalized cell lines, and clinical data to facilitate gene discovery in ALS, http://ccr.coriell.org/Sections/Collections/NINDS/?SsId=10 [149]. This resource currently maintains samples and associated phenotypic data from thousands of motor neuron disease and control subjects.

On the other hand, some perspectives and tendencies in ALS research may quickly turn in accordance with successful results from ongoing clinical trials. The US National Institutes of Health lists 61 clinical trials for ALS currently recruiting volunteers. For instance, it includes studies aimed at assessing the feasibility and security of the intraspinal infusion of autologous bone marrow stem cells for the treatment of ALS patients (ClinicalTrials.gov identifier: NCT00855400). 

To date, our understanding of autoimmune mechanisms in ALS is limited to correlations between manifestation of ALS and immunological alterations, and structural and physiological modifications after treatment with ALS-Abs. However, the autoantigens involved and the underlying mechanisms that induce the generation of such autoantibodies are still not known. The identification of the auto-antigens will allow us to not only develop specific animal models, but also to design rational therapies for specific molecular targets, further characterizing the role of autoimmune mechanisms and developing biochemical tests for early detection of ALS.

## Abbreviations

ALS: Amyotrophic lateral sclerosis
ALS-Abs: Antibodies from ALS patients
[Ca^2+^]_i_: Intracellular calcium concentration
COX-2: Cyclooxygenase-2
EAGMD: Experimental autoimmune gray matter disease
EAMND: Experimental autoimmune motor neuron disease
ELISA: Enzyme-linked immunosorbent assay
ER: Endoplasmic reticulum
HLA: Human leukocyte antigen
IgG: Immunoglobulin G
IP_3_R: Inositol 1,4,5-trisphosphate receptor
IVIG: Intravenous immunoglobulins
MCP-1: Monocyte chemotactic protein-1
M-CSF: Macrophage-colony stimulating factor
MEEPs: Miniature end-plate potentials
MFMN: Multifocal motor neuropathy
MMP-9: Metalloproteinase-9
NMJ: Neuromuscular junction
SOD1: Superoxide dismutase 1
TLI: Total lymphoid irradiation
VDCC: Voltage-dependent calcium channel.
